# A comparison study of optimal and suboptimal intervention policies for gene regulatory networks in the presence of uncertainty

**DOI:** 10.1186/1687-4153-2014-6

**Published:** 2014-04-03

**Authors:** Mohammadmahdi R Yousefi, Edward R Dougherty

**Affiliations:** 1 Department of Electrical and Computer Engineering, The Ohio State University, Columbus, OH 43210, USA; 2 Center for Bioinformatics and Genomic Systems Engineering, Department of Electrical and Computer Engineering, Texas A & M University, College Station, TX 77843, USA

**Keywords:** Optimal intervention, Markovian gene regulatory networks, Probabilistic Boolean networks; Uncertainty; Prior knowledge; Bayesian control

## Abstract

Perfect knowledge of the underlying state transition probabilities is necessary for designing an optimal intervention strategy for a given Markovian genetic regulatory network. However, in many practical situations, the complex nature of the network and/or identification costs limit the availability of such perfect knowledge. To address this difficulty, we propose to take a Bayesian approach and represent the system of interest as an uncertainty class of several models, each assigned some probability, which reflects our prior knowledge about the system. We define the objective function to be the expected cost relative to the probability distribution over the uncertainty class and formulate an optimal Bayesian robust intervention policy minimizing this cost function. The resulting policy may not be optimal for a fixed element within the uncertainty class, but it is optimal when averaged across the uncertainly class. Furthermore, starting from a prior probability distribution over the uncertainty class and collecting samples from the process over time, one can update the prior distribution to a posterior and find the corresponding optimal Bayesian robust policy relative to the posterior distribution. Therefore, the optimal intervention policy is essentially nonstationary and adaptive.

## Introduction

A fundamental problem of translational genomics is to develop optimal therapeutic methods in the context of genetic regulatory networks (GRNs) [1]. Most previous studies rely on perfect knowledge regarding the state transition rules of the network; however, when dealing with biological systems such as cancer cells, owing to their intrinsic complexity, little is known about how they respond to various stimuli or how they function under certain conditions. Moreover, if there exists any knowledge regarding their functioning, it is usually marginal and insufficient to provide a perfect understanding of the full system. To address uncertainty, one can construct an uncertainty class of models, each representing the system of interest to some extent, and optimize an objective function across the entire uncertainty class. In this way, success in therapeutic applications is fundamentally bound to the degree of *robustness* of the designed intervention method.

Markovian dynamical networks, especially probabilistic Boolean networks (PBNs) [2], have been the main framework in which to study intervention methods due to their ability to model randomness that is intrinsic to the interactions among genes or gene products. The stochastic state transition rules of any PBN can be characterized by a corresponding Markov chain with known transition probability matrix (TPM) [3]. Markov decision processes (MDPs), on the other hand, are a standard framework for characterizing optimal intervention strategies. Many GRN optimization problems have been formulated in the context of MDPs - for instance - infinite-horizon control [4], constrained intervention [5], optimal intervention in asynchronous GRNs [6], optimal intervention when there are random-length responses to drug intervention [7], and optimal intervention to achieve the maximal beneficial shift in the steady-state distribution [8]. Herein, PBNs will be our choice of reference model for GRNs.

The first efforts to address robustness in the design of intervention policies for PBNs assumed that the errors made during data extraction, discretization, gene selection and network generation introduce a mismatch between the PBN model and the actual GRN [9,10]. Therefore, uncertainties manifest themselves in the entries of the TPM. A *minimax* approach was taken in which robust intervention policies were formulated by minimizing the worst-case performance across the uncertainty class [9]. Thus, the resulting policies were typically conservative. To avoid the detrimental effects of extreme, but rare, states on minimax design and motivated by the results of Bayesian robust filter design [11], the authors in [10] adopted a Bayesian approach whereby the optimal intervention policy depends on the prior probability distribution over the uncertainty class of networks. Constructing a collection of optimal policies, each being optimal for a member of the uncertainty class, the goal was to pick a single policy from this collection that minimizes the average performance relative to the prior distribution. The corresponding policy provides a *model-constrained robust* (MCR) policy. It was noted that this model-constrained policy may not yield the best average performance among all possible policies (we will later define the set of all possible policies for this problem). The authors also considered a class of *globally robust* (GR) policies, which are designed optimally only for a centrality parameter, such as the mean or median, to represent the mass of the uncertainty distribution.

Since [10] was concerned only with stationary policies, it did not consider the possibility of finding nonstationary policies under a Bayesian updating framework, where state transitions observed from the system are used directly to enrich the prior knowledge regarding the uncertainty class. The resulting nonstationary intervention policy, which we refer to it as the *optimal Bayesian robust* (OBR) policy, is our main interest in the present paper. As our main optimization criterion, we use the expected total discounted cost in the long run. This choice is motivated by the practical implications of discounted cost in the context of medical treatment, where the discounting factor emphasizes that obtaining good treatment outcomes at an earlier stage is favored over later stages.

Since the early development of MDPs, it was recognized that when dealing with a real-world problem it seldom happens that the decision maker is provided with the full knowledge of the TPM, but rather some prior information often expressed in a probabilistic manner. Taking a Bayesian approach, an optimal control policy may exist in the expected value sense specifying the best choice of control action in each state. Since the decision maker’s state of knowledge about the underlying true process evolves in time as the process continues, the best choice of control action at each state might also evolve. Because the observations are acquired through a controlled process (a control action is taken at every stage of the process), the optimal policy derived through the Bayesian framework may not necessarily ever coincide with a policy that is optimal for the true state of nature. In fact, frequently, the optimal policy is not *self-optimizing*[12]; rather, optimal control will provide the best trade-off between exploration rewards and immediate costs.

Bellman [13] considered a special case of this problem - the two-armed bandit problem with discounted cost - and later used the term *adaptive control* for control processes with incompletely known transition probabilities. He suggested transforming the problem into an equivalent dynamic program with completely known transition laws for which the state now constitutes both the physical state of the process and an *information* state summarizing the past history of the observed state transitions from the process [14]. This new state is referred to as the *hyperstate*. Along this line of research, authors in [15-17] developed the theory of the OBR policy for Markov chains with uncertainty in their transition probabilities, where there is a clear notion of optimality defined with respect to all possible scenarios within the uncertainty class. This approach is in contrast with the MCR methodology because the resulting policy may not be optimal for any member of the uncertainty class but it yields the best performance when averaged over the entire uncertainty class.

Following the methodology proposed in [17] and assuming that the prior probability distribution of a random TPM belongs to a conjugate family of distributions which are closed under consecutive observations, one can formulate a set of functional equations, similar to those of fully known controlled Markov chains, and use a method of successive approximation to find the unique set of solutions to these equations. In this paper, we adopt this approach for the robust intervention of Markovian GRNs and provide a simulation study demonstrating the performance of OBR policies compared with several suboptimal methods, such as MCR and two variations of GR policies, when applied to synthetic PBNs with various structural properties and parameters, as well as to a mutated mammalian cell cycle network.

The paper is organized as follows. First, we give an overview of controlled PBNs and review the nominal MDP problem where the TPMs of the underlying Markov chain are completely known. We then formulate the OBR policy for PBNs with uncertainty in their TPMs and provide the dynamic programming solution to this optimization problem. We demonstrate a conjugate family of probability distributions over the uncertainty class where each row of the random TPM follows a Dirichlet distribution with certain parameters. Assuming that the rows are independent, the posterior probability distribution will again be a Dirichlet distribution with updated parameters. This provides a compact representation of the dynamic programming equation and facilitates the computations involved in the optimization problem. Several related suboptimal policies are also discussed in detail. Finally, we provide simulation results over both synthetic and real networks, comparing the performance of different design strategies discussed in this paper.

## Methods

### Controlled PBNs

PBNs constitute a broad class of stochastic models for transcriptional regulatory networks. Their construction takes into account several random factors, including effects of latent variables, involved in the dynamical genetic regulation [3]. The backbone of every PBN is laid upon a collection of Boolean networks (BNs) [18]. A BN is composed of a set of *n* nodes, *V*={*v*^1^,*v*^2^,…,*v*^*n*^} (representing expression level of genes *g*^1^,*g*^2^,…,*g*^*n*^ or their products) and a list of Boolean functions *F*={*f*^1^,*f*^2^,…,*f*^*n*^} describing the functional relationships between the nodes. We restrict ourselves to binary BNs, where we assume that each node takes on value of 0, corresponding to an unexpressed (OFF) gene and 1, corresponding to an expressed (ON) gene. This definition extends directly to any finitely discrete-valued nodes. The Boolean function $f^{i}:{\left\{0,1\right\}}^{j_{i}}\to \{0,1\}$ determines the value of node *i* at time *k*+1 given the value of its predictor nodes at time *k* by $v_{k+1}^{i}=f^{i}(v_{k}^{i1},v_{k}^{i2},\ldots ,v_{k}^{ij_{i}})$, where $\left\{v^{i1},v^{i2},\ldots ,v^{ij_{i}}\right\}$ is the *predictor set* of node *v*^*i*^. In a BN, all nodes are assumed to update their values synchronously according to *F*. The dynamics of a BN are completely determined by its state transition diagram composed of 2^*n*^ states. Each state corresponds to a vector $v_{k}=(v_{k}^{1},v_{k}^{2},\ldots ,v_{k}^{n})$ known as the *gene activity profile* (GAP) of the BN at time *k*. To make our analysis more straightforward, we will replace each GAP, **v**_*k*_, with its decimal equivalent denoted by $x_{k}=1+\overset{n}{\underset{i=1}{\sum }}2^{n-i}v_{k}^{i}$, where $x_{k}\in S=\{1,\ldots ,2^{n}\}$ for all *k*.

A PBN is fully characterized by the same set of *n* nodes, *V*, and a set of *m* constituent BNs, **F**={*F*^1^,*F*^2^,…,*F*^*m*^}, called *contexts*, a selection probability vector *R*={*r*^1^,*r*^2^,…,*r*^*m*^} over **F** (*r*^*i*^≥0 for *i*=1,…,*m* and $\overset{m}{\underset{i=1}{\sum }}r^{i}=1$), a network switching probability *q*>0, and a random gene perturbation probability *p*≥0. At any updating epoch, depending on the value of a random variable *ξ*∈{0,1}, with *P*(*ξ*=1)=*q*, one of two mutually exclusive events will occur. If *ξ*=0 then the values of all nodes are updated synchronously according to an operative constituent BN; if *ξ*=1 then another operative BN, *F*^*l*^∈**F**, is randomly selected with probability *r*^*l*^, and the values of the nodes are updated accordingly. The current BN may be selected consecutively when a switch is called for [1]. PBNs also admit random gene perturbations where the current state of each node in the network can be randomly flipped with probability *p*.

A PBN is said to be *context-sensitive* if *q*<1; otherwise, a PBN is called *instantaneously random*. The number of states in a context-sensitive PBN is *m*2^*n*^, whereas the state transition diagram of an instantaneously random PBN is composed of the same 2^*n*^ states in . It is shown in [19] that averaging over the various contexts, relative to *R*, reduces the transition probabilities of a context-sensitive PBN to an instantaneously random PBN with identical parameters. PBNs with only one constituent BN, i.e., *m*=1, are called BNs with perturbation and are of particular interest in some applications [8,20]. For the sake of simplicity and reducing the computational time, we will focus only on instantaneously random PBNs.

Since the nature of transitions from one state to another in a PBN is stochastic and has the Markov property, we can model any PBN by an equivalent homogeneous Markov chain, whose states are members of  and the TPM of this Markov chain can be calculated as described in [19]. We denote the TPM of an instantaneously random PBN by  and let $\left\{Z_{k}\in S,k=0,1,\ldots \right\}$ be the stochastic process of the state transitions for this PBN. Originating from state i∈S, the successor state j∈S is selected randomly according to the transition probability $P_{\text{ij}}=P(Z_{k+1}=j|Z_{k}=i)$, the (*i*,*j*) element of the TPM. For every i∈S, the transition probability vector $\left(P_{i1},P_{i2},\ldots ,P_{i|S|}\right)$ is a stochastic vector such that $P_{\text{ij}}\geq 0$ and $\underset{j\in S}{\sum }P_{\text{ij}}=1$ for every i∈S. Random gene perturbation guarantees the ergodicity of the equivalent Markov chain, resulting in a unique invariant measure equal to its limiting distribution.

To model the effect of interventions, we assume that PBNs admit an external control input, *A*, from a set of possible inputs signals, , that determines a specific type of intervention on a set of *control genes*. It is common to assume that the control input is binary, i.e., A={0,1}, where *A*=0 indicates no-intervention and *A*=1 indicates that the expression level of a single control gene, *g*^*c*^ (or equivalently *v*^*c*^), for a given *c*∈{1,2,…,*n*}, should be flipped. For this control scheme, *A*=0 does not alter the TPM of the original uncontrolled PBN. However, assuming that the network is in state *i*, the action *A*=1 replaces the row corresponding to this state by the row that corresponds to the state ĩ, where the binary representation of ĩ is the same as *i* except *v*^*c*^ being flipped. The effect of this binary control scheme on any PBN can be easily generalized to more than one control gene with more than two control actions; in this paper, we only consider the binary control scheme.

Let $\left\{(Z_{k},A_{k}),Z_{k}\in S,A_{k}\in A,k=0,1,\ldots \right\}$ denote the stochastic process of a state-action pair. The law of motion for the controlled network, with binary external control, is represented by a matrix P(a) with its (*i*,*j*) element defined as

(1) $$\begin{array}{ll} P_{\text{ij}}(a) & =P(Z_{k+1}=j|Z_{k}=i,A_{k}=a)\\ & =\left\{\begin{array}{c} P_{\text{ij}},\;\;\text{if}\;\;a=0,\\ P_{\mathrm{ĩj}},\;\;\text{if}\;\;a=1. \end{array}\right) \end{array}$$

$$P_{\text{ij}}(a)$$

is the probability of going to state j∈S at time *k*+1 from state i∈S, while taking action a∈A, at time *k*. By this construction, it is clear that the controlled TPM, P(a), can be calculated directly from .

### The nominal problem

External intervention in the context of Markovian networks refers to a class of sequential decision making problems in which actions are taken at discrete time units to alter the dynamics of the underlying GRN. It is usually assumed that the decision maker can observe the state evolution of the network at consecutive time epochs *k*=0,1,…,*N*, where the *horizonN* may be finite or infinite. At each *k*, upon observing the state, the decision maker chooses an action from  that will subsequently alter the dynamics of the network. Hence, the stochastic movement of the GRN from one state to another is completely characterized based on the current state and action taken at this state by (1).

Associated with each state and action, there is an immediate cost function g:S×A×S→R to be accrued until the next decision epoch, which we assume is nonnegative and bounded. This cost may reflect the degree of desirability of different states and/or the cost of intervention that is applied. Whenever the process moves from state *i* to *j* under action *a*, a known cost *g*_*i* *j*_(*a*) is incurred. We also assume that *λ*∈(0,1) is the discount factor reflecting the present value of the future cost. An *intervention policy*, denoted by *μ*, is a prescription for taking actions from the set  at each point *k* in time. In general, one can allow a policy for taking an action at time *k* to be a mapping from the entire history of the process up to time *k* to the action space. This mapping need not be deterministic; on the contrary, it might involve a random mechanism that is a function of the history. However, for the problem we consider, there exists a deterministic policy that is optimal. We denote the set of all admissible policies by . The TPM , initial state *Z*_0_=*i*, and any given policy *μ*={*μ*_0_,*μ*_1_,…} in  determine a unique probability measure, $P_{i}^{\mu }$, over the space of all trajectories of states and actions, which correspondingly defines the stochastic processes *Z*_*k*_ and *A*_*k*_ of the states and actions for the controlled network [12]. In the nominal optimization problem, we desire an intervention policy μ∈ℳ such that the objective function

(2) $$J_{P}^{\mu }(i)=\underset{N\to \infty }{\lim}E_{i}^{\mu }\left\{\overset{N-1}{\underset{k=0}{\sum }}\lambda^{k}g_{Z_{k}Z_{k+1}}(A_{k})\right\},$$

is minimized, i.e., $J_{P}^{*}(i)=\underset{\mu \in ℳ}{\min}J_{P}^{\mu }(i)$ for all i∈S. In the above equation, $E_{i}^{\mu }$ denotes expectation relative to the probability measure $P_{i}^{\mu }$.

This optimization problem is usually solved by formulating a set of simultaneous functional equations and a mapping TJ:S→R, obtained by applying the dynamic programming mapping to any function J:S→R, for all i∈S defined by

(3) $$(\text{TJ})(i)=\underset{a\in A}{\min}\left\{\underset{j\in S}{\sum }P_{\text{ij}}(a)g_{\text{ij}}(a)+\lambda \underset{j\in S}{\sum }P_{\text{ij}}(a)J(j)\right\}.$$

The optimal cost function *J*^∗^ uniquely satisfies the above functional equation, i.e., it is the fixed point of the mapping *T*. One can determine the optimal policy with the help of convergence, optimality, and uniqueness theorems for the solution, proven in [21]. These results furnish an iterative method for successive approximation of the optimal cost function, which in turn gives the optimal intervention policy. It can be further shown that the optimal intervention policy belongs to the class of *stationary deterministic* policies, meaning that *μ*_*k*_=*μ* for all *k* and μ:S→A is a single-valued mapping from states to actions.

### OBR intervention policy

In many real-world intervention scenarios, perfect knowledge regarding  may be unavailable or very expensive to acquire. Therefore, we resort to a probabilistic characterization of the elements of  and optimize relative to this uncertainty. Our results in this section are mainly derived from the Bayesian treatment of MDPs by [17]. Let

(4) $$\begin{array}{ll} \Omega & =\left\{P:P\;\text{is}\;|S|\times |S|,P_{\text{ij}}\geq 0,\right)\\ & \;\;\;\left(\underset{j\in S}{\sum }P_{\text{ij}}=1\;\text{for all}i,j\in S\right\}, \end{array}$$

denote the set of all valid uncontrolled TPMs. The uncertainty about the random matrix  is characterized by the prior probability density π(P) over the set *Ω*. Given π(P) and some initial state *i*, we define

(5) $$J^{\mu }(i,\pi )=\underset{N\to \infty }{\lim}E_{i,\pi }^{\mu }\left\{\overset{N-1}{\underset{k=0}{\sum }}\lambda^{k}g_{Z_{k}Z_{k+1}}(A_{k})\right\},$$

where the expectation is taken not only with respect to the random behavior of the state-action stochastic process but also with respect to the random choice of  according to its prior distribution, π(P). The goal is to find an optimal policy *μ*^∗^ such that (5) is minimized for any i∈S and any prior distribution *π*, i.e., $\mu^{*}={\text{argmin}}_{\mu \in ℳ}J^{\mu }(i,\pi )$. We denote the optimal cost by *J*^∗^(*i*,*π*).

Suppose that we could find optimal intervention policies for every element of *Ω*. Letting $J_{P}^{*}(i)$ denote the optimal cost for any P∈Ω and i∈S and assuming that the optimal cost *J*^∗^(*i*,*π*) exists, we have $E_{\pi }[\;J_{P}^{*}(i)]\leq J^{*}(i,\pi )$ for all i∈S and any *π*. In other words, $E_{\pi }[\;J_{P}^{*}(i)]$ is the best that could be achieved if we were to optimize for every element of the uncertainty class for fixed *i* and *π*.

Since at every stage of the problem an observation is made immediately after taking an action, we can utilize this additional information and update the prior distribution to a posterior distribution as the process proceeds in time. Therefore, we can treat π(P) as an additional state and call (*i*,*π*) the hyperstate of the process. From this point of view, we seek an intervention policy that minimizes the total expected discounted cost when the process starts from a hyperstate (*i*,*π*). Suppose the true, but unknown, TPM is $\hat{P}$. At time 0, the initial state *z*_0_ is known and  is distributed according to *π*. Based on *z*_0_ and *π*, the controller chooses an action *a*_0_ according to some intervention policy. Based on $\left(z_{0},a_{0},\hat{P}\right)$ the new state *z*_1_ is realized according to the probability transition rule ${\hat{P}}_{z_{0}z_{1}}(a_{0})$ and a cost $g_{z_{0}z_{1}}(a_{0})$ is incurred. Based on (*z*_0_,*π*,*a*_0_,*z*_1_), the controller chooses an action *a*_1_ according to some (possibly another) intervention policy and so on [12]. Although the number of states in  and actions in  are finite, the space of all possible hyperstates is essentially uncountable. Therefore, finding an optimal intervention policy which provides a mapping from the space of hyperstates to the space of actions in a sense similar to the nominal case is rather difficult. However, as we will see, it is possible to find an optimal action for a fixed initial hyperstate using an equivalent dynamic program.

#### Dynamic programming solution

We assume that the rows of  are mutually independent. Note that this assumption might not hold true for a large class of problems; however, the analysis becomes overwhelmingly complicated if one is willing to relax this assumption. The posterior probability density of , when the process moves from state *i* to state *j* under control *a*, is found via Bayes’ rule:

(6) $$\pi^{\prime }(P;i,a,j)=\left\{\begin{array}{cc} cP_{\text{ij}}\pi (P), & \;\;\text{if}\;\;a=0,\\ c^{\prime }P_{\mathrm{ĩj}}\pi (P), & \;\;\text{if}\;\;a=1, \end{array}\right)$$

where *c* and *c*^′^ are normalizing constants depending on *i*, *a*, and *j*. Under the sequence of events described above, Martin [17] showed that the minimum expected discounted cost over an infinite period, *N*=*∞*, exists and formulated an equivalent dynamic program with a set of simultaneous functional equations. The dynamic programming operator *T*, similar to (3) but now with the hyperstate (*i*,*π*), takes the following form:

(7) $$\begin{array}{ll} \left(\text{TJ})(i,\pi (P)\right) & =\underset{a\in A}{\min}\left\{\underset{j\in S}{\sum }{\bar{P}}_{\text{ij}}(a)g_{\text{ij}}(a)\right)\\ & \;\left(+\lambda \underset{j\in S}{\sum }{\bar{P}}_{\text{ij}}(a)J(j,\pi^{\prime }(P;i,a,j))\right\}, \end{array}$$

for all i∈S, where ${\bar{P}}_{\text{ij}}(a)=E[\;P_{\text{ij}}(a)]$ with respect to the prior probability density function *π*. It is shown in [17] that there exists a unique bounded set of optimal costs *J*^∗^ satisfying

$$\begin{array}{lll} J^{*}(i,\pi (P)) & =\underset{a\in A}{\min}\left\{\underset{j\in S}{\sum }{\bar{P}}_{\text{ij}}(a)g_{\text{ij}}(a)\right)\; & \;\\ & \;\left(+\lambda \underset{j\in S}{\sum }{\bar{P}}_{\text{ij}}(a)J^{*}(j,\pi^{\prime }(P;i,a,j))\right\},\; & \; \end{array}$$

which is the fixed point of the operator *T*. Since the space of all possible hyperstates (*i*,*π*) is uncountable, construction of an optimal intervention policy for all (*i*,*π*), except for some special cases, may not be feasible. However, given that the process starts at (*i*,*π*), the minimization argument in the above equation yields an optimal action to take only for the current hyperstate.

The difficulty in solving (7), which makes it more complicated than (3), is that the total expected discounted cost when different actions are taken now involves the difference in expected immediate costs and the expected difference in future costs due to being in different states at the next period as well as the effect of different information states resulting from these actions [22]. It should be noted that since the decision maker’s knowledge regarding the uncertainty about  evolves with each transition, the intervention policy will also evolve over time. In a sense, the optimal policy will adapt, implying that stationary optimal policies as defined for the nominal problem do not exist. The optimal nonstationary intervention policy derived through the process discussed above is referred to as the OBR policy.

#### Special case: independent Dirichlet priors

Suppose that both prior and posterior distributions belong to the same family of distributions, i.e., they are conjugate distributions. Then, instead of dealing with prior and posterior at every stage of the problem, we will only need to keep track of the *hyperparameters* of the prior/posterior distributions. A special case of the families of distributions closed under consecutive observations is the Dirichlet distribution, which is the conjugate prior of the multinomial distribution.

Let the initial state *z*_0_ be known and **z**_*n*_=(*z*_0_,*z*_1_,*z*_2_,…,*z*_*n*_) represent a sample path of *n* independent transitions recorded from the network under the influence of an intervention policy. Then the posterior probability density of , $\pi^{\prime }(P)$, can be found using Bayes’ rule:

(8) $$\pi^{\prime }(P)\propto \pi (P)\underset{i\in S}{\prod }\underset{j\in S}{\prod }{\left(P_{\text{ij}}\right)}^{\beta_{\text{ij}}},$$

where *β*_*i* *j*_ denotes the number of transitions in **z**_*n*_ from state *i* to state *j*. The right product in (8) is called the *likelihood function* and the constant of proportionality can be found by normalizing the integral of $\pi^{\prime }(P)$ over *Ω* to 1. Note that although the transitions made in **z**_*n*_ result from an intervention policy, we have formulated the likelihood function only in terms of the elements of  (and not P(a)). This is a consequence of our particular intervention model, where we can substitute for $P_{\text{ij}}(a)$ with $P_{\mathrm{ĩj}}$ whenever *a*=1 as shown in (1). To be more precise, we have $\beta_{\text{ij}}=\beta_{\text{ij}}(0)+\beta_{\mathrm{ĩj}}(1)$, where *β*_*i* *j*_(*a*) is the number of transitions in **z**_*n*_ from state *i* to state *j* under control *a*.

For a fixed state *i*, a transition to state *j* is an outcome of a multinomial sampling distribution with parameters $\left\{P_{i1},P_{i2},\ldots ,P_{i|S|}\right\}$ constituting the standard (|S|−1)-simplex. As stated in the beginning of this section, the conjugate prior for the multinomial distribution is given by the Dirichlet distribution. By the independence assumption imposed on the rows of , one can write the prior for  as

(9) $$\pi (P)=c(\alpha )\underset{i\in S}{\prod }\underset{\in S}{\prod }{\left(P_{\text{ij}}\right)}^{\alpha_{\text{ij}}-1},$$

where *α*_*i* *j*_>0 and *α*= [ *α*_*i* *j*_] is the hyperparameter matrix with the rows arranged in the same manner as . The constant of proportionality is given by

(10) $$c(\alpha )=\underset{i\in S}{\prod }\frac{\Gamma \left(\underset{j\in S}{\sum }\alpha_{\text{ij}}\right)}{\underset{j\in S}{\prod }\Gamma (\alpha_{\text{ij}})},$$

where *Γ* is the gamma function. The uniform prior distribution is obtained if *α*_*i* *j*_=1 for all i,j∈S. As we increase a specific *α*_*i* *j*_, it is as if we bias the posterior distribution on the corresponding element of  with some transition samples before ever observing any samples. It can be verified that

(11) $$E[\;P_{\text{ij}}]=\frac{\alpha_{\text{ij}}}{\underset{l\in S}{\sum }\alpha_{\text{il}}}={\bar{P}}_{\text{ij}},$$

and

$$\text{var}[\;P_{\text{ij}}]=\frac{{\bar{P}}_{\text{ij}}(1-{\bar{P}}_{\text{ij}})}{\underset{l\in S}{\sum }\alpha_{\text{il}}+1}.$$

We also have the following theorem, which is due to Martin [17].

##### 

**Theorem 1**. *Let**have a probability density function given in (9) and (10) with the hyperparameter matrix α* and suppose that a sample with a transition count matrix *β*= [ *β*_*i* *j*_] *is observed. Then the posterior probability density function of**will have the same form as in (9) and (10), but with the hyperparameter matrix α*+*β*.

Assuming *α* as the hyperparameter representing π(P) and using Theorem 1, one can rewrite Equation 7 as

$$\begin{array}{lll} \left(\text{TJ})(i,\alpha \right) & =\underset{a\in A}{\min}\left\{\underset{j\in S}{\sum }{\bar{P}}_{\text{ij}}(a)g_{\text{ij}}(a)\right)\; & \;\\ & \;\left(+\lambda \underset{j\in S}{\sum }{\bar{P}}_{\text{ij}}(a)J(j,\alpha +\gamma )\right\},\; & \; \end{array}$$

where *γ* is a matrix of all zeros except *γ*_*i* *j*_=1 if *a*=0 or $\gamma_{\mathrm{ĩj}}=1$ if *a*=1, and

$${\bar{P}}_{\text{ij}}(a)=\left\{\begin{array}{cc} {\bar{P}}_{\text{ij}}, & \;\;\text{if}\;\;a=0,\\ {\bar{P}}_{\mathrm{ĩj}}, & \;\;\text{if}\;\;a=1. \end{array}\right)$$

The optimal cost *J*^∗^(*i*,*α*) is defined by

$$J^{*}(i,\alpha )=\underset{\mu \in ℳ}{\min}J^{\mu }(i,\alpha ),$$

for a given i∈S and prior hyperparameter *α*. Taking an approach based on the method of successive approximation, let *J*_*k*_(*i*,*α*) for *k*=0,1,… be defined recursively for all i∈S and any valid hyperparameter matrix *α* by

(12) $$\begin{array}{lll} J_{k+1}(i,\alpha ) & =\underset{a\in A}{\min}\left\{\underset{j\in S}{\sum }{\bar{P}}_{\text{ij}}(a)g_{\text{ij}}(a)\right)\; & \;\\ & \;\left(+\lambda \underset{j\in S}{\sum }{\bar{P}}_{\text{ij}}(a)J_{k}(j,\alpha +\gamma )\right\},\; \end{array}$$

with {*J*_0_(*i*,*α*)} as a set of bounded initial functions. Under some mild conditions, the sequence of functions {*J*_*k*_(*i*,*α*)} converges monotonically to the optimal solution *J*^∗^(*i*,*α*) for any i∈S and uniformly for all valid *α*[17]. Faster rates of convergence can be achieved for smaller values of *λ*. Assuming that the method of successive approximation converges in *K* steps, then for a specific value of (*i*,*α*), one needs to evaluate ${\left(|A|\times |S|\right)}^{K}$ terminal values necessary for the computation of *J*^∗^(*i*,*α*). Therefore, to minimize computational time, we restrict ourselves to small values for *λ* and *K*. Once the successive approximation converges, an action *a*^∗^ that minimizes the RHS of (12) is optimal.

**Figure 1:**
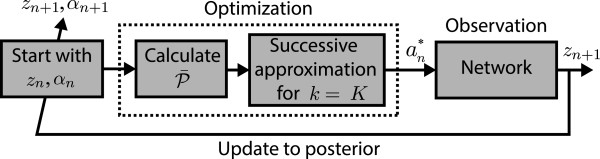
**Optimization procedure for an OBR policy.** We start with a hyperstate (*z*_*n*_,*α*_*n*_). We calculate $\bar{P}$ using *α*_*n*_ and utilize the successive approximation method for a fixed *K* to find an optimal action $a_{n}^{*}$. We then apply the action $a_{n}^{*}$ to the network and let it transition from state *z*_*n*_, or $\tilde{z_{n}}$ depending on the optimal action, to a new random state *z*_*n*+1_ according to $\hat{P}$. We incorporate the new observation into our prior knowledge and update the hyperparameter matrix to *α*_*n*+1_ by incrementing the entry at (*z*_*n*_,*z*_*n*+1_) or $\left({\tilde{z}}_{n},z_{n+1}\right)$ of the hyperparameter matrix *α*_*n*_ by 1. We repeat the entire optimization procedure, but now with the new hyperstate (*z*_*n*+1_,*α*_*n*+1_).

The intervention policy optimally adapts to the consecutive observations as follows: we start with an initial hyperstate (*z*_0_,*α*_0_), with *α*_0_ reflecting our prior knowledge regarding the unknown network (or equivalently ). We can calculate $\bar{P}$ using (11) with respect to *α*_0_ and utilize the successive approximation method in (12) for a fixed *K* to find an optimal action *a*^∗^. We then apply the action *a*^∗^ to the network and let it transition from state *z*_0_, or $\tilde{z_{0}}$ depending on the optimal action, to a new random state *z*_1_ according to $\hat{P}$. We incorporate the new observation into our prior knowledge and update the hyperparameter matrix to *α*_1_ by incrementing the entry at (*z*_0_,*z*_1_) or $\left({\tilde{z}}_{0},z_{1}\right)$ of the hyperparameter matrix *α*_0_ by 1. We repeat the entire optimization procedure, but now with the new hyperstate (*z*_1_,*α*_1_), etc. A schematic diagram of this procedure is demonstrated in Figure 1.

The extreme computational complexity of finding the OBR intervention policy for MDPs with large state-space poses a major obstacle when dealing with real-world problems. It is relatively straightforward to implement the procedure described above for networks with three or four genes. However, for larger networks, one should resort either to clever ways of indexing all possible transitions, such as hash tables or a branch-and-bound algorithm, or to approximation methods, such as reinforcement learning. See [12,22,23] for more details. An alternative approach, as we will demonstrate, is to implement suboptimal methods that, in general, have acceptable performance. Yet another potential approach to circumvent the explosion of the space of all hyperstates is to reduce the size of the uncertainty class. For example, we can assume that some rows of the underlying TPM are perfectly known and uncertainty is only on some other rows, with the implication that the regulatory network is partially known. We will leave the analysis of such approaches to future research.

### Suboptimal intervention policies

Besides the OBR policy, three suboptimal policies are of particular interest: *MCR*, *GR*, and *adaptive GR* (AGR). Similar to the previous section, let  be random, having a probability density π(P) over the set of valid TPMs, *Ω*, defined in (4).

Let ${ℳ}_{\text{MCR}}$ denote the set of all policies that are optimal for some element P∈Ω. Each policy in ${ℳ}_{\text{MCR}}$ is stationary and deterministic (each corresponds to a problem with known TPM). Because *Ω* is uncountable and there exits a finite number of stationary deterministic policies, one might find policies that are optimal for many elements of *Ω*. Assuming that the initial state *Z*_0_ is randomly distributed according to some probability distribution *η*, the policy *μ*_MCR_ yields the minimum cost, which is defined by

(13) $$J_{\text{MCR}}(i)=\underset{\mu \in {ℳ}_{\text{MCR}}}{\min}E_{\pi }\left[E_{\eta }\left[J_{P}^{\mu }(Z_{0})\right]\right],$$

where $J_{P}^{\mu }(Z_{0})$ is defined in (2) for any fixed *Z*_0_ and . Since we are limiting ourselves to policies in ${ℳ}_{\text{MCR}}$, it is seldom the case that a single policy minimizes $E_{\pi }[\;J_{P}^{\mu }(Z_{0})]$ for all $Z_{0}\in S$. Hence, we take the expected value of $J_{P}^{\mu }(Z_{0})$ with respect to *η* in (13) as a single value representing the expected cost. The resulting MCR intervention policy is therefore fixed for a given prior distribution in the sense that it will not adapt to the observed transitions.

We define the GR policy as the minimizing argument for the optimization problem given by $J_{\text{GR}}(i)=\underset{\mu \in ℳ}{\min}J_{\bar{P}}^{\mu }(i)$, for all i∈S, where $\bar{P}\in \Omega$ is the mean of the uncertainty class *Ω* with respect to the prior distribution *π*. The optimization method presented for the nominal problem can be readily applied. Hence, the resulting policy, *μ*_GR_, is stationary and deterministic. In the case of independent Dirichlet priors, $\bar{P}$ is given by Equation 11. Here we are considering the mean as an estimate for unknown . However, one can use any other estimate of  and find the optimal policy in a similar fashion. Similar to the MCR policy, this intervention method is also fixed for a given prior distribution and it will not adapt to the observed transitions.

The AGR policy is similar to the GR policy in the sense that it is optimal for the mean of the uncertainty class *Ω*. However, instead of taking the mean with respect to the prior distribution *π* and using the same policy for the entire process, we update *π* to a posterior *π*^′^, defined in (6), whenever a transition is made and calculate the mean of *Ω* with respect to *π*^′^. Since the posterior evolves as we observe more and more transitions, the AGR policy also evolves - therefore, the name adaptive. We denote the cost and the corresponding policy resulting from this procedure, for any initial hyperstate (*i*,*π*), by *J*_AGR_(*i*,*π*) and *μ*_AGR_, respectively. In the case of independent Dirichlet priors, we can simply replace *π* with *α*.

## Results

In this section, we provide a comparison study on the performance of optimal and suboptimal policies based on simulations on synthetically generated PBNs and a real network. Since we implement the method of successive approximation to calculate *μ*_OBR_, we restrict ourselves to synthetic networks with *n*=3 genes. Given that, as we will show, *μ*_AGR_ yields very similar performance compared to the optimal policy, we can implement *μ*_AGR_ for networks of larger size and use it as the baseline for comparison with other suboptimal policies, keeping in mind that the optimal policy should and will outperform any suboptimal method.

### Synthetic networks

We first consider randomly generated PBNs with *n*=3 genes and *m*=3 equally likely constituent BNs (total number of states being 8) with the maximum number of predictors for each node set to 2 (*j*_*i*_≤2 for all *i*∈{1,2,…,*n*}). The *bias* of a randomly generated PBN is the probability that each of its Boolean regulatory functions takes on the value 1 in its truth table. We assume that the bias is taken randomly from a beta distribution with mean 0.5 and standard deviation 0.01. The gene perturbation probability is assumed to be *p*=0.001. In the context of gene regulation, there are some genes associated with phenotypes (typically undesirable ones). We refer to these genes as *target genes* and our goal in controlling the network is to push the dynamics of these genes away from undesirable states towards desirable ones. Once the set of target genes is identified, one can partition the state space  into subsets of desirable and undesirable states, denoted by  and , respectively. In our synthetic network simulations, we choose the control and target genes to be the least and most significant bits in the binary representation of states, respectively, and assume that downregulation of the target gene is undesirable. As for the discount factor and the immediate cost function *g*_*i* *j*_(*a*), we set *λ*=0.2 and

(14) $$g_{\text{ij}}(a)=\left\{\begin{array}{c} 2.1,\;\text{if}\;\;j\in U\;\;\text{and}\;\;a=1,\\ 2.0,\;\text{if}\;\;j\in U\;\;\text{and}\;\;a=0,\\ 0.1,\;\text{if}\;\;j\in D\;\;\text{and}\;\;a=1,\\ 0,\;\text{otherwise}, \end{array}\right)$$

the interpretation being that a cost will be incurred if the future state in undesirable or there is an intervention in the network.

To design an OBR policy for a given network, we need to assign the prior probability distribution to the set *Ω*. As discussed earlier, independent Dirichlet priors parameterized by *α* constitute a natural choice for this application. Therefore, we only need to assign values to *α*. The choice of prior hyperparameters plays a crucial role in the design of an optimal policy: the tighter the prior around the true, but unknown, TPM $\hat{P}$, the closer the OBR cost is to that of $\hat{P}$. Since our synthetic networks are generated randomly and not according to some biologically motivated GRN, it would be difficult to assign prior probabilities for individual networks. Therefore, we use the randomly generated PBNs themselves for this purpose and perturb and scale the elements of the TPMs via the *ε*-contamination method.

A random PBN, $\hat{P}$, is first generated. This network will serve as the true, but unknown, PBN. Then a contamination matrix  of the same size (|S|×|S|) is generated, where each row is sampled uniformly from the |S−1|-simplex. Note that  is a valid TPM. We now define the hyperparameter matrix *α* by

(15) $$\alpha =\kappa \left((1-\varepsilon )\hat{P}+\varepsilon Q\right),$$

where *κ*>0 controls the tightness of the prior around the true PBN and *ε*∈[0,1] controls the level of contamination. For networks with three genes, we assume that *ε*=0.1 and demonstrate the effect of *κ* on the performance of intervention policies.

We generate 500 random PBNs, denoted by $\left\{N^{l}\right\}$ for *l*=1 to 500, for each set of parameters and calculate their TPMs, denoted by $\left\{{\hat{P}}^{l}\right\}$. These networks will serve as the ground-truth for our simulation study. For a given pair of *κ* and *ε*, we then construct hyperparameter matrices, denoted by {*α*^*l*^}, using (15), each corresponding to a random network. To compare the performance of different intervention policies, for each randomly generated network $N^{l}$, we take a Monte Carlo approach and generate 500 random TPMs, denoted by $\left\{{\hat{P}}^{l,l^{\prime }}\right\}$ for *l*^′^=1 to 500, from the *α*^*l*^-parameterized independent Dirichlet priors. The set $\left\{{\hat{P}}^{l,l^{\prime }}\right\}$ will essentially represent *Ω* and the prior distribution.

To design and evaluate the performance of *μ*_MCR_ for each random PBN $N^{l}$, we proceed as follows: We find the optimal intervention policy for each ${\hat{P}}^{l,l^{\prime }}$, apply this policy to every element in the set $\left\{{\hat{P}}^{l,l^{\prime }}\right\}$, and calculate the average over all equally likely initial states, $Z_{0}\in S$, of the infinite-horizon expected discounted cost using (2) for that element. The expected performance of the each policy optimal for ${\hat{P}}^{l,l^{\prime }}$, relative to the prior distribution, can be computed by taking the average of the resulting costs over all ${\hat{P}}^{l,l^{\prime }}$. We repeat this procedure for every element of $\left\{{\hat{P}}^{l,l^{\prime }}\right\}$ and declare a policy MCR if it yields the minimum expected performance. We denote the expected cost function for a random PBN $N^{l}$ obtained via an MCR policy by $J_{\text{MCR}}^{l}$.

Finding *μ*_GR_ for each PBN $N^{l}$, on the other hand, is easier and it requires only the value of the hyperparameter *α*^*l*^. Once found, the performance of this policy is evaluated by applying it to all elements of $\left\{{\hat{P}}^{l,l^{\prime }}\right\}$ and taking the average of the resulting costs. Similar to the MCR policy, we assume that the initial states are equally likely and calculate the average over all possible initial states. We denote the expected cost function corresponding to the GR policy derived for $N^{l}$ by $J_{\text{GR}}^{l}$.

To quantify the performance of the OBR policy for each random PBN $N^{l}$, we directly evaluate the cost function defined in (5) relative to the independent Dirichlet prior distribution, *π*^*l*^, parameterized by *α*^*l*^. This is accomplished using the sample set of 500 random TPMs, $\left\{{\hat{P}}^{l,l^{\prime }}\right\}$. Starting from a hyperstate and a TPM ${\hat{P}}^{l,l^{\prime }}$, we derive an optimal action from (12) using the method of successive approximations with *K*=5 and some initial cost function. We then observe a transition according to ${\hat{P}}^{l,l^{\prime }}$ and find the incurred discounted immediate cost according to (14), depending on the new observed state and the optimal action just taken. We update our prior hyperparameter and carry out the optimization problem again, but now with the updated hyperparameter and the recently observed state, and accumulate the newly incurred discounted immediate cost. We iterate this for seven epochs, thus observing seven different hyperstates for a sampling path, and record the total accumulated discounted cost over this period. We then repeat this entire process, for the same ${\hat{P}}^{l,l^{\prime }}$ for 100 iterations (although the same TPM is used, different sampling paths will result due to random transitions), and take the average of all 100 total accumulated discounted cost values. This will represent the cost associated with ${\hat{P}}^{l,l^{\prime }}$ and the initial state. We implement a similar procedure for all initial states (assuming all equally likely) and all elements of $\left\{{\hat{P}}^{l,l^{\prime }}\right\}$ and take the average of the resulting costs, yielding the expected optimal cost, *E*_*η*_[*J*^∗^(*Z*_0_,*π*^*l*^)], with respect to the uniform probability distribution *η* over the initial states in . Since we use the same hyperparameter *α*^*l*^ in our Monte Carlo simulation for a given random PBN $N^{l}$, we denote the expected optimal cost obtained from a OBR policy by $J_{\text{OBR}}^{l}$.

We take a similar approach for evaluating the performance of *μ*_AGR_. Instead of using the method of successive approximations at every epoch, we use the current value of the hyperparameter to calculate the mean of *Ω* and use this to find the optimal action to take at that hyperstate. Every other step of the process is essentially the same to those of the OBR policy. We denote the expected optimal cost obtained from this policy by $J_{\text{AGR}}^{l}$.

We also evaluate three other cost functions for each PBN $N^{l}$: $J_{\text{LB}}^{l}:=E_{\pi }\left[E_{\eta }[J_{P}^{*}(Z_{0})]\right]$, $J_{T}^{l}:=E_{\eta }[J_{{\hat{P}}^{l}}^{*}(Z_{0})]$, and $J_{\text{ET}}^{l}:=E_{\pi }\left[E_{\eta }[J_{P}^{l}(Z_{0})]\right],$ where $J_{P}^{l}$ is the cost of applying an optimal intervention policy corresponding to ${\hat{P}}^{l}$ to an element  of *Ω*. The first cost function, $J_{\text{LB}}^{l}$, is a lower bound on the performance of the OBR policy, $J_{\text{BA}}^{l}$. The second cost function, $J_{T}^{l}$, corresponds to the cost of applying an optimal intervention policy as if we knew the true network, ${\hat{P}}^{l}$, to the true network itself. The third cost function, $J_{\text{ET}}^{l}$, is the expected cost, relative to the prior, of applying an intervention policy that is optimal for the true network. We can calculate these cost functions assuming that *Ω* and the prior distribution *π*^*l*^ are represented by the set $\left\{{\hat{P}}^{l,l^{\prime }}\right\}$ corresponding to each PBN $N^{l}$.

All cost functions discussed above are defined relative to a given random PBN $N^{l}$. Since we have 500 such networks, for each parameter value, we report the average performance across all random networks and provide a statistical comparison on the performance of different intervention policies. The results are presented in Table 1. As seen in the table, the optimal policy performance, in the average sense, is consistently better than all suboptimal policies. The closest performance to the optimal method is achieved by the AGR policy, which is not surprising, since this policy adapts to the process over time by updating the prior distribution to a posterior distribution and optimizes with respect to the mean of the posterior.

**Table 1:** **Average costs across all 500 randomly generated PBNs with *n *** **=3 genes and *ε *** **=0 *. *** **1**

	$E[J_{\text{LB}}^{l}]$	$E[J_{T}^{l}]$	$E[J_{\text{ET}}^{l}]$	$E[J_{\text{MCR}}^{l}]$	$E[J_{\text{GR}}^{l}]$	$E[J_{\text{AGR}}^{l}]$	$E[J_{\text{OBR}}^{l}]$
*κ*=0.1	0.7626	1.0803	1.0998	1.0948	1.0991	1.0816	1.0812
*κ*=1.0	0.8078	1.0296	1.0531	1.0520	1.0526	1.0458	1.0457
*κ*=5.0	0.9417	1.0209	1.0525	1.0518	1.0513	1.0502	1.0501

As it has been reported in the previous studies [7,8,24], the performance of an optimal policy might not significantly exceed those of suboptimal policies when averaged across random PBNs; nonetheless, there are networks for which the optimal policy notably outperforms the suboptimal ones. To demonstrate this, we use the difference between the optimal and suboptimal costs to quantify the gain made by implementing an optimal policy. We define *percent decrease* by

$$\Delta_{\circ }^{l}=100\times \frac{J_{\circ }^{l}-J_{∙}^{l}}{J_{\circ }^{l}},$$

where $J_{\circ }^{l}$ and $J_{∙}^{l}$ denote two different intervention policies. Since PBNs are randomly generated, $\Delta_{\circ }^{l}$ will also be a random variable with a probability distribution. We estimate the complementary cumulative distribution function (CCDF) of this distribution for different values of $\Delta_{\circ }^{l}$ using its empirical distribution function.

**Figure 2:**
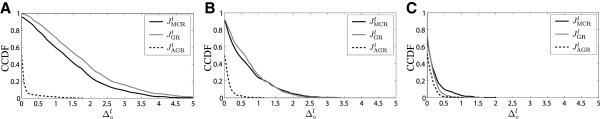
**Empirical CCDF of**$\Delta_{\circ }^{l}$** for different intervention policies across randomly generated PBNs with three genes.(A)***κ*=0.1. **(B)***κ*=1.0. **(C)***κ*=5.0.

For networks with three genes, we assume that $J_{∙}^{l}=J_{\text{OBR}}^{l}$ and $J_{\circ }^{l}$ is any suboptimal policy. Figure 2 shows the empirical CCDF of $\Delta_{\circ }^{l}$ for 500 random PBNs for different values of *κ* and different intervention policies. The graphs illustrate that as the prior distribution gets tighter around the true TPM by increasing *κ*, the difference between the optimal and suboptimal policies vanishes. Again, the best performance among the suboptimal policies is achieved by $J_{\text{AGR}}^{l}$.

As suggested by these results, we may use the suboptimal AGR policy instead of an optimal method for larger networks without a significant lose of optimality. For this purpose, we carry out a similar set of simulations with 500 randomly generated PBNs of size *n*=4 genes. We assume that each PBN consists of *m*=3 equally likely constituent BNs with the maximum number of predictors for each node set to 2, the total number of states being 16. The network bias is drawn randomly from a beta distribution with mean 0.5 and standard deviation 0.01. The gene perturbation probability is *p*=0.001. We generate prior distributions using (15) for each network and different parameter values for *κ* and *ε*. To model *Ω*, we draw 5,000 random TPMs from each prior distribution. We assume that each random sampling path has length 10 and set the discounting factor *λ* to 0.2. We emulate different sampling paths during the calculation of $J_{\text{AGR}}^{l}$ by repeating the entire process for each randomly generated TPM for 1,000 iterations and take the average. The results averaged over 500 random PBNs are presented in Table 2. The AGR policy yields the best performance relative to other suboptimal policies.

**Table 2:** **Average costs across all 500 randomly generated PBNs with *n=4 * genes**

	$E[J_{\text{LB}}^{l}]$	$E[J_{T}^{l}]$	$E[J_{\text{ET}}^{l}]$	$E[J_{\text{MCR}}^{l}]$	$E[J_{\text{GR}}^{l}]$	$E[J_{\text{AGR}}^{l}]$
(*κ*,*ε*)=(0.1,0.0)	0.7559	1.0878	1.0869	1.0856	1.0869	1.0773
(*κ*,*ε*)=(1.0,0.0)	0.8702	1.0888	1.0888	1.0918	1.0888	1.0854
(*κ*,*ε*)=(5.0,0.0)	0.9510	1.0579	1.0578	1.0612	1.0578	1.0572
(*κ*,*ε*)=(0.1,0.1)	0.7711	1.1099	1.1260	1.1248	1.1258	1.1156
(*κ*,*ε*)=(1.0,0.1)	0.8722	1.1106	1.1278	1.1314	1.1276	1.1236
(*κ*,*ε*)=(5.0,0.1)	0.9714	1.0826	1.1011	1.1049	1.1009	1.1002
(*κ*,*ε*)=(0.1,0.25)	0.7177	1.0796	1.1289	1.1234	1.1248	1.1133
(*κ*,*ε*)=(1.0,0.25)	0.8307	1.0853	1.1348	1.1325	1.1305	1.1257
(*κ*,*ε*)=(5.0,0.25)	0.9729	1.0629	1.1178	1.1157	1.1137	1.1130

**Figure 3:**
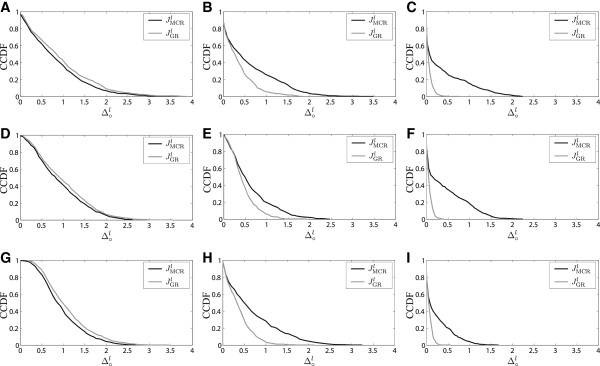
**Empirical CCDF of**$\Delta_{\circ }^{l}$** for different suboptimal intervention policies across randomly generated PBNs with four genes.(A)** (*κ*,*ε*)=(0.1,0.0). **(B)** (*κ*,*ε*)=(1.0,0.0). **(C)** (*κ*,*ε*)=(5.0,0.0). **(D)** (*κ*,*ε*)=(0.1,0.1). **(E)** (*κ*,*ε*)=(1.0,0.1). **(F)** (*κ*,*ε*)=(5.0,0.1). **(G)** (*κ*,*ε*)=(0.1,0.25). **(H)** (*κ*,*ε*)=(1.0,0.25). **(I)** (*κ*,*ε*)=(5.0,0.25).

We graph the empirical CCDF of $\Delta_{\circ }^{l}$ for these networks in Figure 3 for different values of the pair (*κ*,*ε*) and different suboptimal policies. Here, we have that $J_{∙}^{l}=J_{\text{AGR}}^{l}$ and $J_{\circ }^{l}$ are any other suboptimal policy. Similar to networks with three genes, as the prior distributions get more concentrated around the true parameters, the difference between these suboptimal policies gets smaller and smaller. However, it can be seen that the GR policy outperforms MCR for larger *κ*, which could be due to the fact that GR and AGR policies differ very little when the effect of observations on the posterior distribution is dominated by the prior hyperparameters.

### Real network

We construct a PBN corresponding to a reduced network from a mutated mammalian cell cycle network proposed in [25]. The original GRN is a BN with ten genes. Three key genes in the model are Cyclin D (CycD), retinoblastoma (Rb), and p27, where cell division is coordinated with the overall growth of the organism through extracellular signals controlling the activation of CycD in the cell. A proposed mutation for this network is that p27 can never be activated (always OFF), creating a situation where both CycD and Rb might be inactive [25]. Under these conditions, the cell can cycle in the absence of any growth factor, thereby causing undesirable proliferation. Table 3 lists the Boolean functions for this real network.

**Table 3:** Boolean regulatory functions of a mutated mammalian cell cycle

**Gene**	**Node**	**Predictor functions**
CycD	*v*_1_	Extracellular signal
Rb	*v*_2_	$\left(\bar{v_{1}}\wedge \bar{v_{4}}\wedge \bar{v_{5}}\wedge \bar{v_{9}}\right)$
E2F	*v*_3_	$\left(\bar{v_{2}}\wedge \bar{v_{5}}\wedge \bar{v_{9}}\right)$
CycE	*v*_4_	$\left(v_{3}\wedge \bar{v_{2}}\right)$
CycA	*v*_5_	$\left(v_{3}\wedge \bar{v_{2}}\wedge \bar{v_{6}}\wedge (\bar{v_{7}\wedge v_{8}}))\vee (v_{5}\wedge \bar{v_{2}}\wedge \bar{v_{6}}\wedge (\bar{v_{7}\wedge v_{8}})\right)$
Cdc20	*v*_6_	*v*_9_
Cdh1	*v*_7_	$(\bar{v_{5}}\wedge \bar{v_{9}})\vee v_{6}$
UbcH10	*v*_8_	$\bar{v_{7}}\vee (v_{7}\wedge v_{8}\wedge (v_{6}\vee v_{5}\vee v_{9}))$
CycB	*v*_9_	$\left(\bar{v_{6}}\wedge \bar{v_{7}}\right)$

Since the size of the network is too large for the Bayesian treatment, we need to first reduce the number of genes to a more manageable size while preserving important dynamical properties of the network. We have implemented the methodology proposed in [26] and reduced the size of the network to the five genes shown in Table 4. Even for a network of this size, finding the OBR policy is computationally too expensive. Therefore, we only report results for suboptimal policies.

**Table 4:** Boolean regulatory functions of a reduced mutated mammalian cell cycle

**Gene**	**Node**	**Predictor functions**
CycD	*v*_1_	Extracellular signal
Rb	*v*_2_	$\left(\bar{v_{1}}\wedge v_{2}\wedge \bar{v_{3}}\wedge \bar{v_{5}}\right)$
CycA	*v*_3_	$\left(\bar{v_{2}}\wedge v_{3}\wedge \bar{v_{5}})\vee (\bar{v_{2}}\wedge \bar{v_{4}}\vee \bar{v_{5}}\right)$
UbcH10	*v*_4_	$\left(v_{4}\wedge v_{5})\vee (v_{3}\wedge \bar{v_{5}}\right)$
CycB	*v*_5_	$v_{3}\wedge \bar{v_{5}}$

We first construct an instantaneously random PBN for the reduced network. The PBN consists of five genes, CycD, Rb, CycA, UbcH10 and CycB, ordered from the most significant bit to the least significant bit in the binary representation. In the mutated network, depending on the state of the extracellular signal determining the state of CycD as being ON or OFF, we obtain two BNs. These two will serve as two equally likely constituent BNs. It is also assumed that the gene perturbation probability is 0.01. Since cell growth in the absence of growth factors is undesirable, we define undesirable states of the state space to be those for which CycD and Rb are both downregulated. We also choose CycA as the control gene. The immediate cost function is defined similarly to that of the synthetic network simulations (Equation 14). The discounting factor is *λ*=0.2. We calculate the TPM of this network and construct prior hyperparameter matrices *α* using (15) for various pairs of *κ* and *ε*. We generate 10,000 random TPMs from the prior distribution to represent the uncertainty class *Ω*. We also generate 10,000 different sampling paths of length 10 for each random TPM. The total costs are reported in Table 5, where we can see that the results are consistent with those obtained from synthetic networks.

**Table 5:** Total discounted cost of different suboptimal policies for the reduced cell cycle network

	***J***_**LB**_	***J***_**T**_	***J***_**ET**_	***J***_**MCR**_	***J***_**GR**_	***J***_**AGR**_
(*κ*,*ε*)=(0.1,0.0)	0.7507	0.9685	0.9326	0.9465	0.9326	0.9316
(*κ*,*ε*)=(1.0,0.0)	0.4990	0.9685	0.9675	0.9614	0.9675	0.9571
(*κ*,*ε*)=(5.0,0.0)	0.6136	0.9685	0.9658	0.9774	0.9658	0.9605
(*κ*,*ε*)=(0.1,0.1)	0.4501	0.9685	0.9239	0.9268	0.9239	0.9144
(*κ*,*ε*)=(1.0,0.1)	0.5752	0.9685	0.9340	0.9526	0.9340	0.9294
(*κ*,*ε*)=(5.0,0.1)	0.7507	0.9685	0.9326	0.9465	0.9326	0.9316
(*κ*,*ε*)=(0.1,0.25)	0.3885	0.9685	0.8643	0.8674	0.8623	0.8550
(*κ*,*ε*)=(1.0,0.25)	0.5140	0.9685	0.8728	0.8860	0.8730	0.8694
(*κ*,*ε*)=(5.0,0.25)	0.7014	0.9685	0.8864	0.9002	0.8864	0.8861

## Conclusions

Due to the complex nature of Markovian genetic regulatory networks, it is commonplace not to possess accurate knowledge of their parameters. Under the latter assumption, we have treated the system of interest as an uncertainty class of TPMs governed by a prior distribution. The goal is to find a robust intervention policy minimizing the expected infinite-horizon discounted cost relative to the prior distribution. We have taken a Bayesian approach and formulated the intervention policy optimizing this cost, thereby resulting in an intrinsically robust policy. Owing to extreme computational complexity, the resulting OBR policy is, from a practical sense, infeasible. Using only a few genes, we have compared it to several suboptimal polices on synthetically generated PBNs. In this case, although there are PBNs where the OBR policy significantly outperforms the suboptimal AGR policy, on average there is very little difference. Hence, one can feel somewhat comfortable using the AGR policy while losing only negligible performance. Unfortunately, even the AGR policy is computationally burdensome. Hence, when applying it to the mammalian cell cycle network, we are restricted to five genes.

The twin issues of uncertainty and computational complexity are inherent to translational genomics. Here we have examined the problem in the context of therapy, where the uncertainty is relative to network structure. It occurs to also in the other major area of translational genomics, gene-based classification. Whereas here the prior distribution is over an uncertainty class of networks, in classification it is over an uncertainty class of feature-label distributions and one looks for a classifier that is optimal, on average, across that prior distribution [27,28]. There is no doubt, however, that the complexity issue is much graver in the case of dynamical intervention. Hence, much greater effort should be placed on gaining knowledge regarding biochemical pathways and thereby reducing the uncertainty when designing intervention strategies [29]. This means more attention should be paid to classical biological regulatory experiments and less reliance on blind data mining [30].

## Competing interests

The authors declare that they have no competing interests.

## Authors’ contributions

MRY contributed to the main idea, designed and implemented the algorithms, designed and carried out the simulation, analyzed the results, and drafted the manuscript. ERD conceived the study, contributed in the design of the simulation, and revised the manuscript. Both authors read and approved the final manuscript.
